# Health state utilities for skeletal-related events secondary to bone metastases

**DOI:** 10.1007/s10198-012-0443-2

**Published:** 2013-01-25

**Authors:** Louis S. Matza, Karen Chung, Kate Van Brunt, John E. Brazier, Ada Braun, Brooke Currie, Andrew Palsgrove, Evan Davies, Jean-Jacques Body

**Affiliations:** 1 Outcomes Research, United BioSource Corporation, 7101 Wisconsin Avenue, Suite 600, Bethesda, MD 20814 USA; 2 Amgen, Inc., Thousand Oaks, CA USA; 3 Formerly with Outcomes Research, United BioSource Corporation, Bethesda, MD USA; 4 University of Sheffield, Sheffield, UK; 5 Brugmann University Hospital, ULB, Brussels, Belgium

**Keywords:** Utility, Skeletal-related events, Bone metastases, Cancer, Cost-utility, Time trade-off

## Abstract

**Introduction:**

Patients with bone metastases often experience skeletal-related events (SREs). Although cost-utility models are used to examine treatments for metastatic cancer, limited information is available on utilities of SREs. The purpose of this study was to estimate the disutility of four SREs: spinal cord compression, pathological fracture, radiation to bone, and surgery performed to stabilize a bone.

**Methods:**

General population participants from the UK and Canada completed time trade-off (TTO) interviews to assess the utility of health states drafted based on literature review, clinician interviews, and patient interviews. Respondents first rated a health state describing cancer with bone metastases. Then, the SREs were added to this health state.

**Results:**

Interviews were completed with 187 participants (50.8 % male, 80.2 % white). Cancer with bone metastases without an SRE had a mean utility of 0.47 (SD = 0.43) on a standard utility scale (1 = full health, 0 = death). Of the SREs, spinal cord compression was associated with the greatest disutility (i.e., the utility decrease): −0.32 with paralysis and −0.22 without paralysis. Surgery had a disutility of −0.07. Leg, arm, and rib fractures had disutilities of −0.06, −0.04, and −0.03. Two weeks of daily radiation treatment had a disutility of −0.06, while two radiation appointments had the smallest impact on utility (−0.02).

**Conclusion:**

All SREs were associated with statistically significant utility decreases, suggesting a perceived impact on quality of life beyond the impact of cancer with bone metastases. The resulting disutilities may be used in cost-utility models examining treatments to prevent SREs secondary to bone metastases.

## Introduction

The skeleton is one of the most common sites of metastatic disease among adults with breast, prostate, lung, thyroid, and kidney cancers [1, 2]. Bone metastases are associated with poor outcomes, including persistent pain and decreased survival [3]. Furthermore, the spread of cancer cells to the bone results in excessive bone turnover and extensive bone destruction, which leads to skeletal complications, collectively referred to as skeletal-related events (SREs). SREs include pathological fractures, spinal cord compression, surgery to the bone, and radiation to bone [1, 4–7]. In many cases, these SREs can be severely debilitating, resulting in a profound decrease in health-related quality of life (HRQL) [8, 9].

As new treatments are developed for patients who are at risk for SREs, it is important to evaluate their cost-effectiveness in order to demonstrate their value to patients, clinicians, and third-party payers. A cost-utility analysis (CUA) is a type of cost-effectiveness analysis that incorporates the preferences of individuals for different treatment-related outcomes [10, 11]. These preferences are quantified in terms of utilities, which are values representing health status and HRQL on a scale with anchors of 0 representing states as undesirable as being dead and 1 representing full health [12–14]. Because SREs have a significant effect on HRQL [9], they are likely to have an impact on utility and the outcome of a cost-utility analysis.

However, little is known about the disutility (i.e., the utility decrease) associated with individual types of SREs. Although previous research has estimated utilities associated with SREs, these studies have often provided utility estimates for only one or two types of SREs, such as radiation or fracture [9, 15, 16]. Other previous studies have provided utility estimates associated with SREs as a general group, without differentiating among types of SREs [17, 18]. The resulting lack of clarity on the disutility associated with each type of SRE limits the usefulness of these data in economic models. Therefore, the purpose of the current study was to identify the impact of individual types of SREs on health state utility, using time trade-off (TTO) interviews with general population respondents in the UK and Canada.

## Methods

### Development of health states

The health state vignettes representing cancer with bone metastases and SREs were drafted based on literature review and telephone interviews with clinicians and patients. First, a literature review was conducted to identify studies of bone metastases and SREs to ensure that the health states would be grounded in clinical research [3, 9, 19–24]. Information obtained from this literature search was used when drafting structured interview guides for the patient and clinician interviews.

Interviews were conducted with eight medical professionals who had direct experience working with patients who had experienced SREs. Six of the respondents were medical oncologists, one was a radiation oncologist, and one was an acute care nurse practitioner working at a hematology/oncology clinic. Interviews were first conducted to inform health state development. After the health states were drafted, the same respondents were interviewed again to assess the health states’ clarity and accuracy, and minor revisions were made as a result.

Interviews were also conducted with 11 patients recruited from three clinical oncology sites. All patients had breast (six female patients) or prostate (five male patients) cancer, as well as confirmed radiologic evidence of bone metastases. All had experienced at least one of the four SREs within four months of the recruitment screening date. Three patients had experienced a spinal cord compression. Six had experienced pathological fractures (including fractures of the knee, vertebrae, and right clavicle). Ten had received radiation therapy to the bone, and three had surgery to the bone. The interviewers followed a semi-structured interview guide focusing on patients’ experiences with SREs. The language patients used to describe their symptoms and treatment was incorporated into the health states.

Health states were tested in a pilot study with 19 members of the general population (13 female; mean age = 59.0 years) recruited through a newspaper advertisement. The draft health states were administered in a TTO interview to ensure that respondents were able to understand the health states and the interview task. All participants reported that the health states were clear and easy to understand. Some participants suggested minor revisions in formatting and phrasing, and the health states were edited accordingly.

### Final health states administered in the time trade-off interview

The final set of health state vignettes included a “basic health state” (health state A), which was designed to represent a patient with cancer and bone metastases, but without an SRE. This health state included the following statements: “You have cancer that has spread to your bone. In parts of your body where the cancer has spread, the cancer can weaken your bones. You have pain where the cancer has spread to the bone. This pain is aching and present most of the time. The pain increases with movement, and it may interfere with your daily activities. Your cancer requires treatment such as hormone therapy or chemotherapy. Hormone therapy may have side effects such as hot flashes and decreased sex drive. Chemotherapy may have side effects such as hair loss, nausea, and fatigue.”

An additional eight health states (health states B to I) included this basic health state, followed by 4–6 statements describing an SRE, as well as its duration and impact on functioning (the full health state text is presented in the “Appendix”). The eight health states were designed to represent the four SREs: (1) spinal cord compression; (2) pathological fracture; (3) radiation to bone to manage complications such as uncontrolled bone pain or impending fracture; and (4) surgery for bone complications including fractures or potential fractures [3, 19, 21].

Spinal cord compression was represented by two health states describing a compression without (Health State B) and with (C) paralysis because these two types of compression have a dramatically different impact on mobility and quality of life. There were three health states describing pathological fractures of the leg (D), rib (E), and arm (F). These three locations were chosen to represent mild (rib), moderate (arm), and severe (leg) fractures in terms of their impact on pain and mobility. Radiation was represented by two health states describing two possible courses of radiation treatment: daily radiation over a 2-week period (G) and radiation occurring in only two appointments (H). Two treatment courses were included because clinician interviews indicated that duration of radiation treatment tends to vary across geographical regions. Surgery was represented by a health state describing a surgical procedure to stabilize a bone in the leg that had weakened due to the cancer (I). This type of surgery was selected based on clinician input and published literature indicating that pathological fractures tend to occur most frequently in weight-bearing bones such as the femur, and these fractures require surgery to stabilize the bone, reduce pain, and help restore mobility [24].

### Participants in the valuation survey

Because the valuations of health states were intended to yield utilities that may be used in submissions to agencies like National Institute for Health and Clinical Excellence (NICE), most of whom require general population values, the inclusion criteria did not specify any particular clinical characteristics. All participants were required to be at least 18 years old; understand the assessment procedures; and reside in the United Kingdom or Canada.

In the UK, participants were recruited through newspaper advertisements in Edinburgh and London in July 2010. In Canada, participants were recruited through advertisements in Montréal in September 2010 and Toronto in December 2010. In the UK, a total of 592 potential participants responded to the advertisements by leaving a telephone message, and 179 of these were reached for screening to assess whether they met study inclusion criteria (i.e., 179 participants answered the phone when called by project staff). Of the 179 screened participants, all were eligible, 147 were available to be scheduled for interviews, 130 participants attended interviews, and 126 of these participants were able to complete the TTO interview. In Canada, a total of 523 potential participants responded, and 105 were reached for screening. Of the 105 potential participants who were screened, 102 were eligible, 74 were scheduled, 63 attended the interview, and 61 were able to complete the TTO interview.

### Utility interview procedures and scoring

The utility interview began with a visual analogue scale (VAS) that was intended to introduce participants to the health states. Health states were presented to each participant on individual cards, and each was rated relative to anchor states of zero (dead) and 100 (full health). Then, health state utilities were obtained using the TTO method, which has previously been described in detail [25]. The TTO assessments of health state utilities are often conducted using a 10-year time frame, as this time frame was used in the Measurement and Valuation of Health (MVH) study to elicit valuations from the general public for EQ-5D health states [26]. However, other time frames may be used, depending on what is most appropriate for the medical condition under examination. For the current study, a 2-year time frame was used so that the impact of the SREs would be judged within the context of a realistic life expectancy for a patient with advanced cancer and bone metastases.

In the TTO task, participants were first presented with the basic health state (health state A) and offered a choice between spending two years in this health state versus spending varying shorter amounts of time in the full health state, followed by death. After rating Health State A, participants were presented with each of the SRE health states (Health States B–I) in random order. For each of these SRE health states, participants were told to consider a lifespan of two years in Health State A, with the SRE occurring roughly in the middle of the 2-year time period. Respondents were told that the SRE occurred roughly in the middle of the 2-year lifespan in order to avoid potential biases stemming from reluctance to experience the SRE immediately or at the end of one’s life. Participants concluded the TTO task by rating their own current health state.

For each health state rated as preferable to being dead in the TTO task, the utility value is calculated based on the choice in which the respondent is indifferent between *y* months in the health state being evaluated and *x* months in full health (followed by *y*–*x* months dead). The resulting utility estimate (*u*) is calculated by setting to equal the expected value of the two options [1* *x* + 0 * (*y* – *x*) = *u* * *y*], and then rearranging to solve for *u* (*u* = *x*/*y*). In the current study, *y* is two years.

If participants indicated that a health state was worse than dead, the interviewer altered the task so that respondents were offered a choice between immediate death (alternative 1) and a 2-year life span (alternative 2) beginning with varying amounts of time in the health state being rated, followed by full health for the remainder of the two years. For TTO ratings of health states considered worse than death, two scoring approaches have been used in previous studies, as described by Brazier et al. [25]. The first approach, which is based on the choice in which the respondent is indifferent between the two alternatives, yields utilities with a possible range of 0 to −∞. These unbounded negative values have a strong tendency to skew the overall distribution of utility estimates for any health states that are rated as worse than dead by even a small number of respondents. Therefore, the current study used an alternative bounded scoring approach, which is commonly used to avoid highly skewed distributions. This approach limits the range of utilities for health states worse than death so that scores are between 0 and −1. To compute these bounded negative utility values, the current study used the Dolan [46] method as described by Rowen and Brazier [34]. This method uses the formula “−*x*/*t*”, where x is the number of months in full health, and *t* is the total life span of alternative 2 in the TTO choice. In the current study, *t* is 24 months, which is the number of months in the health state being rated plus subsequent months in full health.

The purpose of this study was not only to identify the utilities of various health states, but also to identify the disutility associated with each specific SRE. The disutility of each SRE was calculated as the difference between the utility of the basic health state (metastatic cancer without an SRE) and the utility of the otherwise identical health states with an added SRE. Calculating differences between health states to identify disutilities of specific attributes has been shown to be useful in other utility studies [27, 28].

### RAND-36

The RAND Health Survey 36-item short form (RAND-36) was administered for use in analyses assessing the validity of the utility procedure by comparing the RAND-36 score to the utility score for the participant’s own current health. The RAND-36 consists of 36 items contributing to eight scales: physical functioning, social functioning, role limitations due to physical health problems, role limitations due to emotional problems, pain, mental health, vitality, and general health perceptions [29].

### Data collection and statistical analysis procedures

Interviews were conducted in Edinburgh and London during August 2010, as well as in Montréal and Toronto from September 2010 to January 2011. All procedures and instruments were approved by an independent Institutional Review Board, and all participants provided written informed consent. Statistical analyses were completed using SAS version 8.12 (SAS Institute, Cary, NC). Continuous variables including utilities are summarized in terms of means and standard deviations, and categorical variables such as gender and race are summarized as frequencies and percentages.

The disutility of each SRE was calculated by subtracting the utility of each SRE health state (B–I) from the utility of health state A. This disutility quantifies the impact of the SRE on a 2-year life span, which was the timeframe of the TTO task in the current study. These disutilities may be used to compute the impact of SREs on quality-adjusted life years (QALYs), which are used to quantify outcomes in a cost-utility analysis so that treatments may be compared in terms of cost per QALY gained. The QALY is a general measure of health outcomes that incorporates both quality of life and quantity of life, with quality defined in terms of utilities and quantity defined in terms of years [11, 30–32].

To demonstrate the impact of each type of SRE on QALYs, the overall QALY decrement associated with each SRE was calculated for the combined UK/Canada sample. Because the disutility values in the current study represent the impact of an SRE on a 2-year lifespan, the disutility should be applied throughout a 2-year period of a cost-utility model when modeling a patient who experiences an SRE (i.e., subtracting the disutility from a patient’s utility value for both of these two years). For example, an SRE with a disutility of −0.10 would be applied for two years of the patient’s life, resulting in a total QALY decrement of −0.20 over the 2-year period. Therefore, the total QALY decrement associated with each SRE can be computed by doubling the disutility value resulting from the current TTO task.

Spearman’s correlations between the TTO utilities for current health and the RAND-36 subscale scores were performed to assess validity of the TTO procedure. Correlation coefficients were interpreted based on Cohen’s [33] guidelines suggesting that a coefficient of 0.10–0.29 is small, 0.30–0.49 is moderate, and greater than 0.50 is large. To examine differences among health states, paired t-tests were conducted to assess whether differences between key pairs of health states were statistically significant. Statistical significance was considered to be *p* < 0.05.

## Results

### Sample description

A total of 187 participants completed the utility interview (Table 1). The sample was almost evenly divided between men and women (49.2 % female), and the mean age was 46.1 years. A majority of the sample reported their ethnicity as being white (80.2 %), and 38.5 % reported being currently married. Almost half of the sample were employed full-time (*n* = 82; 43.9 %), and the majority completed a college or university degree (*n* = 115; 61.5 %). When asked to report health conditions, over half the sample reported none (*n* = 113, 60.4 %). The most commonly reported health conditions were depression (*n* = 19, 10.2 %) and arthritis (*n* = 16, 8.6 %). Only 3.2 % of the total sample (*n* = 6) reported that they had a diagnosis of cancer at any time in their lives, and no participants reported cancer that had metastasized to the bone.

**Table 1:** Demographic characteristics

Characteristics	UK (*N* = 126)	Canada (*N* = 61)	Total sample: UK and Canada (*N* = 187)
Age (mean, SD)	45.4 (16.0)	47.7 (9.9)	46.1 (14.3)
Gender (*n*, %)			
Male	65 (51.6 %)	30 (49.2 %)	95 (50.8 %)
Female	61 (48.4 %)	31 (50.8 %)	92 (49.2 %)
Ethnicity (*n*, %)			
White	107 (84.9 %)	43 (70.5 %)	150 (80.2 %)
Black	11 (8.7 %)	6 (9.8 %)	17 (9.1 %)
Asian	3 (2.4 %)	8 (13.1 %)	11 (5.9 %)
Other	5 (4.0 %)	4 (6.6 %)	9 (4.8 %)
Marital status (*n*, %)			
Married	49 (38.9 %)	23 (37.7 %)	72 (38.5 %)
Not married	77 (61.1 %)	38 (62.3 %)	115 (61.5 %)
Employment status (*n*, %)			
Full-time work	46 (36.5 %)	36 (59.0 %)	82 (43.9 %)
Part-time work	34 (27.0 %)	10 (16.4 %)	44 (23.5 %)
Unemployed	10 (7.9 %)	3 (4.9 %)	13 (7.0 %)
Other^a^	36 (28.6 %)	12 (19.7 %)	48 (25.7 %)
Education level (*n*, %)			
Completed college/university degree	82 (65.1 %)	33 (54.1 %)	115 (61.5 %)
Did not complete college/university degree	44 (34.9 %)	28 (45.9 %)	72 (38.5 %)

^a^Other includes homemaker, caregiver, student, retired, and disabled

Demographic characteristics are also presented separately for the UK and Canadian sub-samples (Table 1). There were no statistically significant differences between the two sub-samples in age, gender, marital status, or education level. There was a statistically significant difference in racial/ethnic background (*p* < 0.05). Although both samples were predominantly white, the UK sample had a greater proportion of white participants. Additionally, a greater proportion of the Canadian sample was employed full-time (59.0 vs. 36.5 %; *p* < 0.05).

### Descriptive statistics: utilities

The VAS scores and TTO utilities for all health states are presented in Tables 2 and 3, respectively. In the total sample, the basic health state (A) describing cancer with bone metastases without an SRE had a mean VAS score of 38.5 and a TTO utility of 0.47. For the eight health states that included an SRE (health states B–I), mean VAS scores ranged from 1.0 to 31.7, and mean TTO utilities ranged from 0.15 to 0.45. The *t* tests comparing the UK and Canadian samples found no significant differences in utilities for any of these health states (*p* = 0.46–0.99). The mean utility for respondents’ own current health was 0.94, which is reflective of a healthy general population sample.

**Table 2:** Visual analog scale health state ratings

Health states	UK (*N* = 126)		Canada (*N* = 61)		Total sample: UK and Canada (*N* = 187)	
	Mean	SD	Mean	SD	Mean	SD
A: basic health state (cancer with bone metastases, no SRE)	42.3	25.6	30.6	16.9	38.5	23.7
B: basic HS + spinal cord compression without paralysis	10.5	21.1	2.6	21.3	7.9	21.4
C: basic HS + spinal cord compression with paralysis	2.5	24.0	−2.2	24.0	1.0	24.1
D: basic HS + fracture of the leg	22.0	22.8	11.1	18.3	18.5	22.0
E: basic HS + fracture of the rib	31.0	24.0	18.8	16.6	27.0	22.6
F: basic HS + fracture of the arm	25.4	21.9	15.2	17.7	22.1	21.1
G: basic HS + radiation treatment (2 weeks, 5 appointments per week)	31.1	24.1	18.2	19.3	26.9	23.4
H: basic HS + radiation treatment (2 appointments)	35.7	24.6	23.4	16.4	31.7	22.9
I: basic HS + surgery to stabilize bone	22.9	25.2	11.9	19.6	19.3	24.0
Own current health state	89.6	9.1	85.0	11.0	88.1	9.9

*HS* health state

*SRE* skeletal-related event

* VAS values are on a scale with anchors of 0 representing death and 100 representing full health

**Table 3:** Time trade-off health state utilities

Health states	UK (*N* = 126)		Canada (*N* = 61)		Total sample: UK and Canada (*N* = 187)	
	Mean	SD	Mean	SD	Mean	SD
A: basic health state (cancer with bone metastases, no SRE)	0.47	0.41	0.47	0.45	0.47	0.42
B: basic HS + spinal cord compression without paralysis	0.25	0.50	0.25	0.54	0.25	0.51
C: basic HS + spinal cord compression with paralysis	0.13	0.49	0.19	0.53	0.15	0.50
D: basic HS + fracture of the leg	0.42	0.41	0.40	0.48	0.41	0.43
E: basic HS + fracture of the rib	0.44	0.42	0.43	0.47	0.44	0.43
F: basic HS + fracture of the arm	0.44	0.41	0.43	0.48	0.43	0.43
G: basic HS + radiation treatment (2 weeks, 5 appointments per week)	0.42	0.42	0.41	0.50	0.41	0.45
H: basic HS + radiation treatment (2 appointments)	0.45	0.41	0.45	0.45	0.45	0.42
I: basic HS + surgery to stabilize bone	0.40	0.44	0.39	0.50	0.40	0.46
Own current health state	0.95	0.05	0.92	0.13	0.94	0.09

*HS* health state

*SRE* skeletal-related event

* TTO utilities are on a scale with anchors of 0 representing death and 1 representing full health

The disutility of each SRE was computed by subtracting the utility of each SRE health state from the utility of health state A, which is an otherwise identical health state without an SRE (Table 4). The SRE disutilities ranged from −0.02 for two radiation appointments to −0.32 for spinal cord compression with paralysis. The smallest disutilities were for milder SREs including two radiation appointments (disutility = −0.02), and pathological fractures of the rib and arm (−0.03 and −0.04, respectively). Two weeks of radiation treatment (−0.06), pathological fracture of the leg (−0.06), and surgery (−0.07) were associated with a larger disutility, while spinal cord compression was associated with the greatest disutility of all the SREs (−0.22 without paralysis and −0.32 with paralysis). There were no significant differences in disutilities between the samples in the UK and Canada (*p* = 0.27 to 0.97). These disutilities represent the impact of each SRE on preferences for the 2-year life span in the current TTO task. The total QALY decrement of each individual SRE across the lifespan ranged from −0.05 to −0.63 (Table 4).

**Table 4:** Disutilites and QALY decrease associated with skeletal-related events

Health states representing each SRE	Disutility*							
	UK (*N* = 126)		Canada (*N* = 61)		Total sample: UK and Canada (*N* = 187)		QALY decrement** (*N* = 187)	
	Mean	SD	Mean	SD	Mean	SD	Mean	SD
B: basic HS + spinal cord compression without paralysis	−0.22	0.31	−0.22	0.32	−0.22	0.31	−0.44	0.62
C: basic HS + spinal cord compression with paralysis	−0.34	0.36	−0.28	0.30	−0.32	0.34	−0.63	0.68
D: basic HS + fracture of the leg	−0.05	0.09	−0.07	0.19	−0.06	0.13	−0.11	0.26
E: basic HS + fracture of the rib	−0.03	0.08	−0.04	0.17	−0.03	0.12	−0.06	0.24
F: basic HS + fracture of the arm	−0.03	0.07	−0.04	0.17	−0.04	0.11	−0.07	0.23
G: basic HS + radiation treatment (2 weeks, 5 appointments per week)	−0.05	0.12	−0.06	0.21	−0.06	0.15	−0.11	0.31
H: basic HS + radiation treatment (2 appointments)	−0.02	0.07	−0.02	0.11	−0.02	0.08	−0.05	0.17
I: basic HS + surgery	−0.07	0.17	−0.08	0.21	−0.07	0.18	−0.15	0.37

*SRE* skeletal-related event

*HS* health state

* TTO utilities are on a scale with anchors of 0 representing death and 1 representing full health. Disutilities of each SRE are computed by subtracting health state A (bone metastases without an SRE) from each of the other health states (bone metastases with an SRE)

** The disutility values represent the impact of each SRE on a 2-year lifespan, which was the time horizon of the TTO task in this study. Therefore, the total QALY decrement associated with each SRE was computed by doubling the disutility value resulting from the TTO task

### Correlations between self-reported health status and time trade-off utility of respondent’s own current health

Since this study was conducted with a sample from the general population, most participants had a high TTO utility for their own current health (mean = 0.94; SD = 0.09; mode = 0.96 which was the utility for 135 of the 187 participants). Despite the limited variability in these utilities, all correlations between the TTO utility for participants’ own current health and the scales of the RAND-36 were in the expected direction, ranging from 0.16 to 0.36. Correlations with the following scales were statistically significant: role limitations due to physical health (*r* = 0.24; *p* < 0.01), role limitations due to emotional problems (*r* = 0.24; *p* < 0.05), vitality (*r* = 0.20; *p* < 0.05), social functioning (*r* = 0.28; *p* < 0.01), pain (*r* = 0.23; *p* < 0.05), general health (*r* = 0.36; *p* < 0.001), physical component summary score (*r* = 0.23; *p* < 0.05), and mental component summary score (*r* = 0.20; *p* < 0.05). Overall, these correlations indicate that higher TTO utilities for own participants’ current health were associated with better health status.

### Comparisons among health state utilities

Paired *t* tests in the total sample found that utilities of all SRE health states (B–I) were significantly different from the utility of health state A, which did not include an SRE (*t* = 3.7 to 12.7; all *p* < 0.001). The T-tests were also conducted to examine whether utilities of similar health states were significantly different from each other. The two radiation health states, G (2 weeks) and H (two appointments), had significantly different mean utilities (*t* = −3.6; *p* < 0.001). The two spinal cord compression health states, B (without paralysis) and C (with paralysis), also had significantly different mean utilities (*t* = 7.2; *p* < 0.001). The health states representing pathological fractures of the rib (E) and arm (F) both had significantly higher utilities than health state D representing a leg fracture (*t* = −4.7 and −4.1, respectively; both *p* < 0.001).

## Discussion

The current study provides a more detailed assessment of utilities associated with SREs than has previously been available. Each of the four SREs had an impact on utility, and there were logical statistically significant differences among the health states. For example, respondents differentiated between radiation treatment of different frequencies and among three types of pathological fractures. It has been suggested that differences among health state utilities of at least 0.05 can be considered clinically important [30]. The disutilities of most SREs in the current study exceeded this threshold, indicating that SREs have an important impact on utility. In light of these results, it is recommended that researchers conducting cost-utility models of treatment for bone metastases consider incorporating the disutility associated with SREs. Furthermore, because each of the four SREs appears to be associated with a distinct disutility, they should be quantified individually rather than applying a common disutility value across all SREs.

The utilities followed logical patterns. For example, surgery had a stronger impact on utility than either radiation or pathological fracture, and spinal cord compression was associated with a substantially greater disutility than any other SRE. Furthermore, the health state representing radiation treatment with only two appointments had the smallest disutility. Adding to confidence in the study procedures, the TTO utility for respondents’ own current health was significantly associated with their self-reported health status as represented by most scales of the RAND-36. In sum, the logical pattern of results suggests that respondents understood the TTO task, and the resulting utilities adequately represent their preferences among the health states.

Data collection for the current study was completed first in the UK, followed by the replication in Canada. The Canadian sample was added to provide further support for the utilities and to examine whether the influence of SREs on preferences would vary in a different culture and geographic location. Although VAS ratings for each health state were lower in Canada than in the UK, the TTO utility results were remarkably similar across the two countries. The consistency across the two countries adds to confidence that the current results are reasonable estimates of the disutility of the four SREs.

Although TTO methods yielded logical utilities, it should be noted that other utility assessment methods are possible. The current health states could be rated with generic multi-attribute classification systems such as the EQ-5D, SF-6D, and Health Utilities Index [34]. However, the generic multi-attribute measures have some important limitations. For example, they are unlikely to be sensitive to clinically important aspects of health states that are not directly captured by their limited number of items and response options. Therefore, they may not be detailed enough to reflect the impact of specific medical conditions and treatment attributes, such as those described in the current SRE health states [34–39]. In contrast, the current TTO approach allowed respondents to consider every aspect of the health states when providing their responses. Furthermore, multi-attribute measures have been shown to have ceiling and floor effects, which makes them less sensitive to health states in the upper or lower ends of the utility scale range [25, 35, 37, 40, 41]. Because of these limitations of generic multi-attribute measures, direct utility elicitation methods were considered preferable for assessing the current health states.

Despite the strengths and logical results of this study, several limitations of the study design should be acknowledged. First, vignette-based utility assessment methods are limited because respondents rate health states based on brief descriptions in the vignettes, rather than direct personal experience. Although vignettes for the current study were carefully drafted based on published literature as well as patient and clinician descriptions of each SRE, the accuracy of each utility is limited by the level of detail and clarity of the vignettes. For example, it is possible that participants underestimated the impact of a pathological leg fracture. The mean disutility of 0.06 may not capture the full extent of this painful and potentially debilitating experience. Despite efforts to accurately represent a pathological fracture, it is possible that this health state seemed less aversive to some respondents because a fracture is a familiar experience. Some respondents could have been thinking of their own experiences with bone fractures that healed relatively quickly, instead of considering the full impact of a pathological fracture experienced by a patient with bone metastases. A key advantage of vignette-based utility assessment is that it can be used to identify utilities of specific factors that may be difficult to isolate in a patient sample. Instruments which are designed to derive utilities from patient samples, such as the EQ-5D or Health Utilities Index, may not have items or response options that are sensitive to specific medical conditions, events, or treatment attributes [42]. In contrast, health state vignettes can be designed to focus on any specific aspect of disease or treatment that may be important to capture in a cost-utility model. The extent to which vignette-based utilities would correspond to utilities derived from direct patient experience is not known.

Characteristics of the sample should also be considered when interpreting findings. Reimbursement authorities often prefer that cost-utility analyses use utilities derived from general population respondents to ensure that societal values are represented when making decisions about public funding for medical treatment [38, 43–45]. Therefore, the current study was conducted with a broad sample of respondents regardless of their health status or clinical history. However, the participants were not specifically recruited to be representative of the general population, and it is not known whether preferences for health states would be different in a truly representative sample. In addition, the recruitment strategy involving newspaper and Internet advertisements could have introduced sample selection biases to the extent that some potential participant groups may have less access to these media outlets. Still, the current sample was reasonably diverse with regard to most demographic variables, and there is no reason to believe their values would be systematically different from a nationally representative sample.

Several characteristics of the health states also suggest that results should be interpreted with appropriate caution. First, these health states describe a severe medical condition, and consequently, 48 (25.7 %) of the 187 respondents rated at least one health state as worse than being dead (26.2 % in the UK; 24.6 % in Canada). A concern with TTO methodology is that health states worse than dead are rated in a slightly different procedure than states with positive scores (as described in the methods section), and the resulting negative scores are on a different scale [25]. There are several available approaches for transforming negative utilities so that they are on the same scale as positive utilities, but no consensus has been reached on a most widely accepted method. The transformation approach used in the current study was selected in order to make the values comparable to those from the UK EQ-5D valuation study [46]. New methods are being developed for valuing states worse than dead, but these methods are not yet widely used [34].

Another challenge associated with these health states is that they were developed to capture the disutility of relatively brief events, lasting several months or less. Typical utility assessment methods involving valuation of unchanging health state vignettes would not have been appropriate for assessment of SRE disutilities. For example, the most common time trade-off approach is to value health states lasting ten years without a change. Clearly, SREs such as a fracture cannot be described in an unchanging health state of this duration. To capture the disutilities of SREs in the current study, it was necessary to specify that the events were temporary in order to realistically represent the temporary nature of SREs in the context of cancer with bone metastases. However, because the health states returned to the pre-SRE state after the temporary event, the resulting disutilities do not capture any residual or long-term impact of having an SRE. Some SREs have been shown to be associated with lasting impact, such as increased pain or analgesic use [47]. Therefore, findings may underestimate the impact of these SREs.

When interpreting and using these disutilities for modeling, it is important to remember that they represent the impact of a single SRE on preferences for a two-year lifespan. There are two possible approaches for using these disutility values in a cost-utility analysis. One approach is to apply the disutility for a 2-year period when modeling a patient who experiences an SRE (i.e., subtracting the disutility from a patient’s utility value for these two years). If the disutility value is used in this way, the total QALY decrease across the patient’s lifespan would be double the value of the disutility itself. For example, to model a patient experiencing a spinal cord compression without paralysis, the disutility of −0.22 would be applied for two years of the patient’s life, resulting in a total QALY decrement of −0.44 over the 2-year period. A second, and possibly simpler, approach would be to apply a one-time QALY decrement for each SRE that is experienced, using a value that is double the disutility for the particular SRE. For example, a one-time QALY decrement of −0.44 would be used for patients expressing spinal cord compression without paralysis. These two approaches yield mathematically equivalent results in terms of the impact of SREs on the total number of QALYs and the outcomes of a cost-utility model.

The two-year time frame of the TTO task may also have implications. Although longer time horizons such as ten years are more commonly used for TTO valuation, the two-year time frame was selected to more accurately represent a typical lifespan of patients with bone metastases [48]. The QALY model is based on the assumption that the utility of a health state is the same regardless of how long someone is in the state. There is evidence that utilities may vary based on the time horizon of a TTO procedure [49, 50], although some findings suggest that this variation may be relatively small [51].

Current results provide the disutility of each individual SRE, and it is not clear how these values may change when modeling patients with multiple SREs in a single year. For example, multiple SREs could be combined additively, but this approach may not accurately represent patient experiences. The SREs often occur in sequence, as single patients may experience one SRE followed by another. It is possible that the true impact of two SREs could be greater or less than the sum of the two disutilities, depending on the individual patient and the unique combination of SREs.

Despite the limitations, the current study is a step toward more thorough modeling of treatment for patients with bone metastases. The current disutilities may be used to compute QALY decrements associated with SREs so that the impact of these often debilitating events can be represented in cost-utility analyses. Future research may further examine and refine the disutilities of SREs, especially multiple co-occurring SREs, among patients in clinical trials or among larger nationally representative general population samples.

## Appendix: Health states

### Health state A: basic health state (cancer with bone metastases, no SREs)

- You have cancer that has spread to your bone.
- In parts of your body where the cancer has spread, the cancer can weaken your bones.
- You have pain where the cancer has spread to the bone. This pain is aching and present most of the time.
- The pain increases with movement, and it may interfere with your daily activities.
- Your cancer requires treatment such as hormone therapy or chemotherapy. Hormone therapy may have side effects such as hot flashes and decreased sex drive. Chemotherapy may have side effects such as hair loss, nausea, and fatigue.

### Health state B: basic health state + spinal cord compression without paralysis

- Because your cancer has spread to your spinal column, there is pressure on nerves in your spinal cord that connect to the rest of your body.
- The compression of nerves in your spinal column leads to severe pain in your back. The pain also runs down your legs and increases with abrupt motion, like with coughing or sneezing.
- You have numbness and tingling in your legs.
- You have severe pain, which causes difficulty sleeping, walking, and moving around.
- You sometimes have difficulty controlling bowel and bladder function, leading to occasional incontinence.
- These issues persist for about 3 months.

### Health state C: basic health state + spinal cord compression with paralysis

- Because your cancer has spread to your spinal column, there is pressure on nerves in your spinal cord that connect to the rest of your body.
- Because of the compressed nerves, you are paralyzed below the waist.
- You have serious pain in your back, above the area of paralysis. This pain causes difficulty sleeping.
- You cannot walk or stand by yourself. You require a wheelchair to get around.
- You have persistent difficulty controlling bowel and bladder function, leading to persistent fecal and urinary incontinence.
- These issues persist for about 3 months.

### Health state D: basic health state + pathological fracture of the leg

- Because your cancer has weakened your bone, you have a fracture in your leg resulting from a minor impact.
- The fracture causes extreme, intense pain in your leg. The pain becomes worse with movement and causes difficulty sleeping.
- You cannot walk or put any weight on your leg for the first month.
- Although the pain decreases with treatment, you continue to have some pain and mobility problems for about 3 months.

### Health state E: basic health state + pathological fracture of the Rib

- Because your cancer has weakened your bone, you have a fracture in your rib that occurred spontaneously, without any impact.
- You have moderate pain in the area where the fracture occurred, particularly with movement.
- The fracture causes discomfort when something touches this area of your body.
- Although the pain decreases with treatment, you continue to have some pain for about 3 months.

### Health state F: basic health state + pathological fracture of the arm

- Because your cancer has weakened your bone, you have a fracture in your arm resulting from a minor impact.
- The fracture causes extreme, intense pain in your arm. The pain becomes worse with movement and causes difficulty sleeping.
- You cannot move your arm or lift any weight for the first month.
- Although the pain decreases with treatment, you continue to have some pain and limited use of your arm for about 3 months.

### Health state G: basic health state + radiation treatment (2 weeks, Five appointments per week)

- Because your pain has recently increased, you receive radiation treatment to control cancer cell growth in your bone and to reduce some of your bone pain.
- You attend radiation appointments 5 days per week for 2 weeks.
- Each radiation appointment lasts about 30 min. During the procedure, you lie still and experience no pain (like when getting an x-ray).
- In the area where you receive radiation, your skin becomes irritated, like getting a sunburn. You also feel fatigued after each treatment.

### Health state H: basic health state + radiation treatment (Two Appointments)

- Because your pain has recently increased, you receive radiation treatment to control cancer cell growth in your bone and to reduce some of your bone pain.
- You attend a total of two radiation appointments.
- Each radiation appointment lasts about 30 min. During the procedure, you lie still and experience no pain (like when getting an x-ray).
- In the area where you receive radiation, your skin becomes irritated, like getting a sunburn. You also feel fatigued after the treatment.

### Health state I: basic health state + surgery to stabilize bone

- You undergo surgery to stabilize a bone in your leg that has weakened due to the cancer.
- A metal rod (about 10 cm in length) is inserted into your leg to stabilize the bone and hold it in place. The surgery itself is performed at a hospital and lasts roughly 3 h. You receive general anesthetic for the surgery.
- You remain in the hospital for about 10 days while you recover from surgery. After you leave the hospital, you continue to attend frequent physical therapy (i.e., physiotherapy) appointments.
- For the first few weeks after the surgery, you need help getting up and moving around.
- As you recover from the surgery, you continue to have some pain and mobility problems for about 3 months.

## References

[1] Coleman RE (1997). Skeletal complications of malignancy. Cancer.

[2] Virk MS, Lieberman JR (2007). Tumor metastasis to bone. Arthritis Res Ther.

[3] Coleman RE (2001). Metastatic bone disease: clinical features, pathophysiology and treatment strategies. Cancer Treat. Rev..

[4] Fizazi K, Carducci M, Smith M, Damiao R, Brown J, Karsh L, Milecki P, Shore N, Rader M, Wang H, Jiang Q, Tadros S, Dansey R, Goessl C (2011). Denosumab versus zoledronic acid for treatment of bone metastases in men with castration-resistant prostate cancer: a randomised, double-blind study. Lancet.

[5] Lipton A, Theriault RL, Hortobagyi GN, Simeone J, Knight RD, Mellars K, Reitsma DJ, Heffernan M, Seaman JJ (2000). Pamidronate prevents skeletal complications and is effective palliative treatment in women with breast carcinoma and osteolytic bone metastases: long term follow-up of two randomized, placebo-controlled trials. Cancer.

[6] Rosen LS, Gordon D, Tchekmedyian NS, Yanagihara R, Hirsh V, Krzakowski M, Pawlicki M, De Souza P, Zheng M, Urbanowitz G, Reitsma D, Seaman J (2004). Long-term efficacy and safety of zoledronic acid in the treatment of skeletal metastases in patients with nonsmall cell lung carcinoma and other solid tumors: a randomized, Phase III, double-blind, placebo-controlled trial. Cancer.

[7] Stopeck AT, Lipton A, Body JJ, Steger GG, Tonkin K, de Boer RH, Lichinitser M, Fujiwara Y, Yardley DA, Viniegra M, Fan M, Jiang Q, Dansey R, Jun S, Braun A (2010). Denosumab compared with zoledronic acid for the treatment of bone metastases in patients with advanced breast cancer: a randomized, double-blind study. J. Clin. Oncol..

[8] Body JJ, Diel IJ, Bell R, Pecherstorfer M, Lichinitser MR, Lazarev AF, Tripathy D, Bergstrom B (2004). Oral ibandronate improves bone pain and preserves quality of life in patients with skeletal metastases due to breast cancer. Pain.

[9] Weinfurt KP, Li Y, Castel LD, Saad F, Timbie JW, Glendenning GA, Schulman KA (2005). The significance of skeletal-related events for the health-related quality of life of patients with metastatic prostate cancer. Ann. Oncol..

[10] Torrance GW (1986). Measurement of health state utilities for economic appraisal. J Health Econ.

[11] Torrance GW, Spilker B (1996). Designing and conducting cost-utility analyses. Quality of Life and Pharmacoeconomics in Clinical Trials.

[12] Feeny D, Barr RD, Furlong W, Torrance GW, Weitzman S, Osaba D (1991). Quality of life of the treatment process in pediatric oncology: an approach to measurement. Effect of Cancer on Quality of Life.

[13] Torrance GW (1987). Utility approach to measuring health-related quality of life. J Chronic Dis.

[14] Torrance GW, Furlong W, Feeny D (2002). Health utility estimation. Expert Rev Pharmacoeconomics Res.

[15] Milne RJ, Heaton-Brown KH, Hansen P, Thomas D, Harvey V, Cubitt A (2006). Quality-of-life valuations of advanced breast cancer by New Zealand women. Pharmacoeconomics.

[16] Reed SD, Radeva JI, Glendenning GA, Saad F, Schulman KA (2004). Cost-effectiveness of zoledronic acid for the prevention of skeletal complications in patients with prostate cancer. J. Urol..

[17] De Cock E, Hutton J, Canney P, Body JJ, Barrett-Lee P, Neary MP, Lewis G (2005). Cost-effectiveness of oral ibandronate compared with intravenous (i.v.) zoledronic acid or i.v. generic pamidronate in breast cancer patients with metastatic bone disease undergoing i.v. chemotherapy. Support. Care Cancer.

[18] Dranitsaris G, Hsu T (1999). Cost utility analysis of prophylactic pamidronate for the prevention of skeletal related events in patients with advanced breast cancer. Support. Care Cancer.

[19] Brown JE, Coleman RE (2002). The present and future role of bisphosphonates in the management of patients with breast cancer. Breast Cancer Res..

[20] Chow E, Hird A, Velikova G, Johnson C, Dewolf L, Bezjak A, Wu J, Shafiq J, Sezer O, Kardamakis D, Linden Yvd, Ma B, Castro M, Arnalot PF, Ahmedzai S, Clemons M, Hoskin P, Yee A, Brundage M, Bottomley A (2009). The European organisation for research and treatment of cancer quality of life questionnaire for patients with bone metastases: the EORTC QLQ-BM22. Eur. J. Cancer.

[21] Clemons M, Dranitsaris G, Cole D, Gainford MC (2006). Too much, too little, too late to start again? Assessing the efficacy of bisphosphonates in patients with bone metastases from breast cancer. Oncologist.

[22] Drudge-Coates L (2006). Skeletal complications and the use of bisphosphonates in metastatic prostate cancer. Int J Palliat Nurs.

[23] Lin A, Ray ME (2006). Targeted and systemic radiotherapy in the treatment of bone metastasis. Cancer Metastasis Rev..

[24] Mundy GR (2002). Metastasis to bone: causes, consequences and therapeutic opportunities. Nat. Rev. Cancer.

[25] Brazier JR, Ratcliffe J, Salomon JA, Tsuchiya A (2007). Measuring and Valuing Health Benefits for Economic Evaluation.

[26] Dolan P, Gudex C, Kind P, Williams A (1996). The time trade-off method: results from a general population study. Health Econ..

[27] Matza LS, Boye KS, Yurgin N, Brewster-Jordan J, Mannix S, Shorr JM, Barber BL (2007). Utilities and disutilities for type 2 diabetes treatment-related attributes. Qual. Life Res..

[28] Boye KS, Matza LS, Walter KN, Van Brunt K, Palsgrove AC, Tynan A (2011). Utilities and disutilities for attributes of injectable treatments for type 2 diabetes. Eur J Health Econ.

[29] Ware JE, Sherbourne CD (1992). The MOS 36-item short-form health survey (SF-36). I. Conceptual framework and item selection. Med. Care.

[30] Feeny D, Fayers P, Hays R (2005). Preference-based measures: utility and quality-adjusted life years. Assessing Quality of Life in Clinical Trials.

[31] Torrance GW (1997). Preferences for health outcomes and cost-utility analysis. Am. J. Manag. care.

[32] Torrance GW, Feeny D (1989). Utilities and quality-adjusted life years. Int. J. Technol. Assess. Health Care.

[33] Cohen J (1988). Statistical Power Analysis for the Behavioral Sciences.

[34] Rowen D, Brazier J, Glied S, Smith PC (2011). Health utility measurement. The Oxford Handbook of Health Economics.

[35] Brazier J, Jones N, Kind P (1993). Testing the validity of the Euroqol and comparing it with the SF-36 health survey questionnaire. Qual. Life Res..

[36] Brazier J, Deverill M, Green C (1999). A review of the use of health status measures in economic evaluation. J. Health Serv. Res. Policy.

[37] Kaarlola A, Pettila V, Kekki P (2004). Performance of two measures of general health-related quality of life, the EQ-5D and the RAND-36 among critically ill patients. Intensive Care Med..

[38] Stein K, Fry A, Round A, Milne R, Brazier J (2005). What value health? A review of health state values used in early technology assessments for NICE. Appl. Health Econ. Health Policy.

[39] Tolley K (2009). What are Health Utilities?.

[40] Brazier J, Roberts J, Tsuchiya A, Busschbach J (2004). A comparison of the EQ-5D and SF-6D across seven patient groups. Health Econ..

[41] Ferreira PL, Ferreira LN, Pereira LN (2008). How consistent are health utility values?. Qual. Life Res..

[42] Garau M, Shah KK, Mason AR, Wang Q, Towse A, Drummond MF (2011). Using QALYs in cancer: a review of the methodological limitations. Pharmacoeconomics.

[43] Brazier J (2008). Valuing health States for use in cost-effectiveness analysis. Pharmacoeconomics.

[44] NICE: Guide to the methods of technology appraisal. In. National Institute for Health and Clinical Excellence, London, (2008) 27905712

[45] PBAC: Guidelines for preparing submissions to PBAC, Version 4.3.2. In. Pharmaceutical Benefits Advisory Committee, (2008)

[46] Dolan P (1997). Modeling valuations for EuroQol health states. Med. Care.

[47] Fallowfield, L., Cleeland, C.S., Body, J.J., Stopeck, A., von Moos, R., Patrick, D.L., Clemons, M., Tonkin, K., Masuda, N., Lipton, A., De Boer, R., Salvagni, S., Tosello, O.C., Ying, W., Braun, A., Cong, Z.: Pain severity and analgesic use associated with Skeletal-related events in patients with advanced breast cancer and bone metastases. Cancer Res (2011: 71 (24 Suppl): Abstract nr P4-13-01)

[48] Coleman RE (2006). Clinical features of metastatic bone disease and risk of skeletal morbidity. Clin. Cancer Res..

[49] Lee GM, Salomon JA, LeBaron CW, Lieu TA (2005). Health-state valuations for pertussis: methods for valuing short-term health states. Health Qual Life Outcomes.

[50] Stiggelbout A, Kiebert G, Kievit J, Leer J, Habbema J, De Haes J (1995). The “utility” of the time trade-off method in cancer patients: feasibility and proportional trade-off. J. Clin. Epidemiol..

[51] Brazier J, Dolan P, Karampela K, Towers I (2006). Does the whole equal the sum of the parts? Patient-assigned utility scores for IBS-related health states and profiles. Health Econ..

